# Integrated transcriptomics and metabolomics confirms the oxidative stress mechanism of hypothermia-induced neuronal necroptosis

**DOI:** 10.1186/s11658-025-00772-0

**Published:** 2025-07-21

**Authors:** Song-Jun Wang, Chao-Long Lu, Fu Zhang, Xue-Tong Dong, Xiao-Rui Su, Jing-Jing Sha, Bin Cong, Xia Liu

**Affiliations:** 1 Hebei Key Laboratory of Forensic Medicine, Collaborative Innovation Center of Forensic Medical Molecular Identification, Research Unit of Digestive Tract Microecosystem Pharmacology and Toxicology, Chinese Academy of Medical Sciences, College of Forensic Medicine, Hebei Medical University, No. 361 Zhong Shan Road, Shijiazhuang, Hebei China; 2 Forensic Pathology Lab, Guangdong Public Security Department, No. 97 Huanghua Road, Guangzhou, 510050 China; 3 Hainan Tropical Forensic Medicine Academician Workstation, Haikou, 571199 Hainan China

**Keywords:** Hypothermia, Death from cold, Cryoinjury, Oxidative stress, Necroptosis, Environmental stress

## Abstract

Abnormal climate change seriously endangers the safety of outdoor work and life, often causing hypothermia-induced coma or death. As the underlying mechanism has not been fully elucidated, a targeted treatment for hypothermia-triggered neuronal injury and forensic pathology indicators of fatal hypothermia are lacking. Herein, we aimed to explore hypothermia-induced changes in gene expression and metabolite profiles of cerebral cortical tissues to elucidate the mechanism of hypothermia-promoted necroptosis of cerebral cortical neurons. Flow cytometry and fluoro-jade C staining showed that low temperature caused necroptosis of cerebral cortical neurons. Transcriptomics identified 244 differential genes between hypothermia-exposed cortical tissue and control tissue. These genes were enriched in tumor necrosis factor (TNF)-α and nuclear factor (NF)-kappa B signaling pathways, as revealed by Kyoto Encyclopedia of Genes and Genomes (KEGG) pathway enrichment analysis. Broadly targeted metabolomics identified 49 differential metabolites with significant differences. *N*-alpha-acetyl-L-arginine, argininosuccinic acid, glutaric acid, and other ornithine cycle-associated metabolites were significantly reduced in the hypothermia-exposed cortical tissue, driving fumaric acid reduction in the tricarboxylic acid (TCA) cycle. In addition, KEGG enrichment analysis showed significant changes in the TCA cycle pathway. A combined transcriptomic and metabolomic analysis uncovered that hypothermia induced oxidative stress through NF-κB activation, caused mitochondrial damage, impaired the ornithine cycle, and, ultimately, induced neuronal necroptosis. Pharmacological inhibition of NF-κB by the SC75741 inhibitor effectively ameliorated hypothermia-triggered necroptosis. In conclusion, our results suggest that the NF-κB transcription factor is a potential marker of hypothermia-induced neuronal necroptosis in the mouse cerebral cortex. In addition, our findings indicate the mechanism of necroptosis in cerebral cortical neurons caused by low temperature, which is beneficial for our understanding of hypothermia-induced coma and death.

**Supplementary information:**

The online version contains supplementary material available at 10.1186/s11658-025-00772-0.

## Highlights

1. Combined transcriptomic and metabolomic analysis reveal key mechanisms by which hypothermia-induced neuronal necroptosis occurs in cerebral cortical tissue.
2. Necroptosis is the main type of hypothermia-induced neuronal death in cerebral cortical tissue.
3. Oxidative stress is the main mechanism by which hypothermia induces neuronal necroptosis in cerebral cortical tissue.
4. NF-κB–inducible nitric oxide synthase (iNOS) signaling pathways are key targets of hypothermia-induced neuronal necroptosis in cerebral cortical tissue.

**Supplementary information:**

The online version contains supplementary material available at 10.1186/s11658-025-00772-0.

## Background

Climate change is one of the most pressing global challenges of modern times [1, 2]. It causes severe convective weather that creates considerable damage to the natural environment and affects the safety of outdoor work and life, often causing hypothermia-induced coma and death. In the USA, approximately 2000 people die each year from weather-related causes, of which 63% are attributed to exposure to excessive or prolonged natural cold [3, 4]. In addition, in less developed countries or regions, low temperatures can be particularly life threatening. Since the mechanisms of hypothermia-induced coma and death have not been fully elucidated, targeted treatment options for hypothermia-induced neuronal injury and forensic pathological identification indicators for death from hypothermia are lacking [5–10].

The cerebral cortex is the surface layer of gray matter of the cerebrum that regulates consciousness, movement, and body temperature. It is also a critical area of hypothermia injury and plays a crucial role in defense against lethal hypothermia injury. Hypothermia leads to decreased metabolism of the central nervous system, aggravated neuronal damage, and central nervous system dysfunction [11–14]. Evidence suggests that pyramidal cells in the cerebral cortex of rats exposed to induced fatal hypothermia are necrotic, with enhanced acidophilic cytoplasm, absent nucleoli, karyolysis, and chromatin fragmentation [15]. Therefore, clarifying the type and mechanism of neuronal death caused by hypothermia should elucidate the unresolved questions related to the outcomes of hypothermia.

Mild and moderate hypothermia exert neuroprotective effects in brain tissue by several mechanisms: reduced oxygen consumption and the production of reactive metabolites (e.g., oxygen free radicals), inhibited synthesis and release of excitatory amino acids, and enhanced activity of superoxide dismutase [16–18]. However, severe hypothermia lowers cardiac output and blood pressure and provokes capillary paralysis, resulting in decreased blood flow. It also elevates red blood cell precipitation, blood viscosity, and hemoglobin oxygen affinity, reducing the oxygen supply in brain tissue [19]. Different degrees of hypothermia alter the internal environment of the body, causing corresponding metabolic and functional disorders [20]. Integrating data from multiple omic levels can reveal the interactions and regulatory relationships between molecules, and identify key molecules and pathways in metabolic and functional disorders [21]. Integrating liver transcriptomic and metabolomic data reveals that energy metabolism and amino acid metabolism are the primary signaling pathways for the response to cold stress in *Pelodiscus sinensis* [22], whereas unsaturated fatty acid metabolism, bile salt transport, vitamin intake, and antioxidant capacity are the main mechanisms causing cold-induced stress damage in pufferfish [23]. However, the combined application of multiomic technologies is rarely reported in the impact of cold stress on the brains of mice, which hinders our understanding of the mechanisms by which cold stress causes neurological damage.

Necroptosis is a form of cell death triggered by tumor necrosis factor (TNF)-α, mediated by a necrosome composed of mixed lineage kinase domain-like proteins (MLKL), receptor-interacting protein kinase 1 (RIPK1), and RIPK3, occurring in the absence of caspase 8. The primary morphological features of necroptosis include organelle swelling, lack of nuclear fragmentation, membrane rupture, and intracellular component leakage [24, 25]. Therapeutic hypothermia can inhibit necrotic apoptosis of neurons in traumatic brain injury [26]; however, severe hypothermia induces necrotic apoptosis in zebrafish [27, 28]. This study aimed to explore hypothermia-induced changes in gene expression and metabolite profiles of cerebral cortical tissues to elucidate the mechanism of hypothermia-promoted necroptosis of cerebral cortical neurons and resolve the scientific issues related to hypothermia-induced coma and death. Our results provide a scientific basis for treating hypothermia-induced neuronal injury and suggest potential molecular markers for forensic pathological identification of death by hypothermia.

## Materials and methods

### Experimental animals

The Animal Care Management Committee of Hebei Medical University approved all animal experiments (approval no. 20223011). A total of 96 male 8-week-old C57BL/6N mice weighing 20 ± 2 g from Beijing Vital River Laboratory Animal Technology Co., Ltd. (Beijing, China) were used in the study. Mice were housed in specific pathogen-free conditions at constant temperature (23 ± 2 ℃) and 50% humidity with a 12 h light–dark cycle.

### Surgical operation and core temperature monitoring

Mice were anesthetized with isoflurane (5% induction, 2–3% maintenance), and their abdominal hair was shaved. The skin was disinfected with iodophor and ethanol, and a longitudinal incision was made along the middle of the abdomen. A sterilized core temperature-monitoring capsule was placed into the abdominal cavity. Mice were subjected to 1 week of adaptive feeding before the experiment.

### Model preparation and sample extraction

Mice were randomly assigned to three groups: control (CON), hypothermia coma (HP90min), and freezing to death (FD). Mice were anesthetized with isoflurane gas, and their hair was shaved, especially on the head, to simulate frigid conditions while working or living outdoors. Mice from the HP90min group were placed in a 2–6 ℃ refrigerator for 90 min and developed hypothermia coma. The mice showed signs of coma such as body curling, loss of righting and writhing reflex, inability to move owing to pricking pain, etc. The FD group mice were exposed to a refrigerator of 2–6 ℃. During the coma period, the electrocardiogram was detected by limb leads, and the electrocardiogram results were used as the basis for judging death. Mice from the CON group and HP90min group were subjected to decapitation under anesthesia. The cerebral cortical tissue was collected for transmission electron microscopy, transcriptomic and extensive targeted metabolomic analysis, and western blotting. The brain was collected for morphological investigation. To further investigate the role of nuclear factor kappa-B (NF-κB) in hypothermia-induced neuronal necroptosis, the specific NF-κB inhibitor SC75741 (1 mg/kg) was administered 1 h before placing the mice in the hypothermia box. SC75741 was dissolved in 10% dimethylsulfoxide (DMSO), 30% Cremophor EL, and 60% phosphate-buffered saline (PBS). SC75741, in a volume of 200 μL, was applied to the mice by intraperitoneal injection [29]. Control animals were treated with the formulation only, containing no compound (placebo).

### Fluoro-jade C (FJC) staining

Brain tissue was sliced into 5-μm-thick sections using a microtome. Brain tissue sections were dewaxed and incubated in 0.06% potassium permanganate solution for 10 min at room temperature on a shaking platform. The slices were incubated in 0.0001% FJC (TR-100-FJT, Biosensis Pty, Ltd., Thebarton, Australia) for 10 min at room temperature and without intense light exposure. The sections were sealed with neutral gum and observed and photographed under a fluorescence microscope.

### Flow cytometry

Cerebral cortical tissue was collected on ice and placed on 180 mesh stainless steel mesh with tweezers. Tissue was passed through the mesh into a centrifuge tube containing precooled PBS and centrifuged at 1000 rpm for 5 min. A single-cell suspension was prepared by adding 100 µL of the binding buffer to each tube. Subsequently, 5 µL of Annexin V/7-AAD was added to each tube and incubated for 15 min at room temperature (RT; 25 °C) in darkness. Before flow cytometry analysis, 400 μL of the binding buffer was added per tube. A flow cytometer (Beckman Coulter FC 500) was used for detection, and Fluorescence Minus One (FMO) controls are employed for setting quadrant gates [30, 31].

### Transmission electron microscopy (TEM)

Observations were done in the cerebral cortex (i.e., neocortical layer, L5). Cerebral cortical tissue was sliced into ultra-thin 50-nm-thick sections and stained with uranium and cobalt, following a standard method. All samples were examined using a TEM microscope (Hitachi HT7800, Japan).

### Metabolome analysis

Mice were assigned to a control and hypothermia coma group with eight mice per group (*n* = 8). Cerebral cortical tissue was collected into 2 mL sterile tubes and stored in liquid nitrogen at −80 °C. After thawing and mashing on ice, a 50 mg tissue sample was mixed with 500 μL of 70% methanol/water (precooled to −20 °C) and swirled for 3 min. Tubes were centrifuged at 12,000 rpm for 10 min at 4 ℃, and 300 μL of supernatant was transferred into a 1.5 mL centrifuge tube. Tubes were refrigerated at −20 ℃ for 30 min and centrifuged at 12,000 rpm at 4 ℃ for 10 min. Subsequently, 200 μL of the supernatant was taken and passed through a protein precipitation plate for liquid chromatography–mass spectrometry (LC–ESI–MS/MS) analysis. The system was provided by Wuhan Maiwei Biotechnology Co., Ltd. (Wuhan, China). Mass spectrometry data were qualitatively analyzed with the Metware database (MWDB) and processed using Analyst 1.6.3 (Sciex, Framingham, MA, USA). The peak intensity of mass spectra corresponding to different concentrations of standard solutions were collected. By substituting the linear equation of the standard curve into the calculation formula, the integral peak area ratio of all tested samples was calculated, and finally the data of the substance in the actual samples were obtained.

### Transcriptome analysis

#### RNA extraction and library construction

Using the principle of random grouping, a control and hypothermia coma group were used as experimental groups, with three samples per group (one sample = cerebral cortical tissue isolated from three experimental mice). Total RNA was extracted using TRIzol reagent as per the manufacturer’s instructions. The purity and quantity of the isolated RNA were determined using a NanoDrop 2000 spectrophotometer (Thermo Scientific, USA), and RNA integrity was assessed using an Agilent 2100 Bioanalyzer (Agilent Technologies, Santa Clara, CA, USA). Transcriptome libraries were constructed using a VAHTS Universal V6 RNA-seq Library Prep Kit, following the manufacturer’s protocol. Shanghai OE Biotechnology Co., LTD (Shanghai, China) performed the transcriptome sequencing and analysis.

#### RNA sequencing and differential gene expression analysis

The libraries were sequenced using the Illumina Novaseq 6000 sequencing platform to generate 150 bp double-end reads. Approximately 48.19 MB raw reads were obtained for each sample and processed with fastp quality control to get clean reads. Hisat2 [32] software was used to align the reads with the reference genome to obtain the positional information of genes, determine the unique sequence characteristics of the sequenced samples, and calculate the gene expression level [33]. Read counts (i.e., the number of aligned reads overlapping exons of each gene) were obtained with HTSeq-count software [34]. Principal component analysis of genes (counts) and mapping were performed using R 3.2.0 to assess biological replicates.

Differentially expressed genes were identified on the basis of |*logFC*|> *1.5* and adjusted *P*-value < *0.05* criteria using DESeq2 software [35]. Hierarchical clustering analysis of these genes was performed to show their expression patterns across distinct groups and samples. A radar map of the top 30 genes was drawn using the R package ggradar to show the expression changes of upregulated or downregulated genes. Gene Ontology [36], Kyoto Encyclopedia of Genes and Genomes (KEGG) [37], Reactome, and WikiPathways pathway enrichment analyses of differentially expressed genes were performed using the clusterProfiler package to screen significantly enriched functional entries. Gene set enrichment analysis (GSEA) was performed [38]. The predefined gene sets were used to rank the genes according to their degree of differential expression in two types of samples and test whether the prespecified gene sets were enriched at the top or the bottom of the ranking table.

### Reverse transcription-quantitative PCR (RT-qPCR)

Total RNA was extracted from 60 mg of cerebral cortical tissue with 1000 µL of TRIzol reagent (Thermo Fisher Scientific, USA), following the manufacturer’s instructions. The quantity of isolated RNA was determined with a NanoDrop ND-1000 spectrophotometer (NanoDrop Technologies, USA), and RNA integrity was assessed using a Bioanalyzer 2100 system (Agilent Technologies). Total RNA was reverse transcribed into cDNA using a PrimerScript RT Kit with gDNA Eraser (Takara Bio Inc.) The synthesized cDNA was quantified with a PrimeScript RT Reagent Kit and TB Green Premix Ex Taq II (Takara Bio Inc.) on an ABI 7500 sequence detection system (Applied Biosystems), following the manufacturer’s protocol. The Ct value of each target was obtained by calculating the arithmetic mean from technical triplicates. The relative expression levels of mRNA relative to the 18S rRNA (i.e., endogenous control) were calculated with the 2 ^−ΔΔCt^ method. Primer sequences used in our study were as follows:

TNF-α forward primer: 5′ CAT CTT CTC AAA ATT CGA GTG ACAA 3′

TNF-α reverse primer: 5′ TGG GAG TAG ACA AGG TAC AAC CC 3′

NF-κB p65 forward primer: 5′ GAA GAA GCG AGA CCT GGAG 3′

NF-κB p65 reverse primer: 5′TCC GGA ACA CAA TGG CCAC 3′

iNOS forward primer: 5′ CAG GAG GAG AGA GAT CCG ATT TA 3′

iNOS reverse primer: 5′ GCA TTA GCA TGG AAG CAA AGA 3′

18S forward primer: 5′ GTA ACC CGT TGA ACC CCA TT 3′

18S reverse primer: 5′ CCA TCC AAT CGG TAG TAG CG 3′

### Immunohistochemistry (IHC)

For IHC, 5-μm-thick paraffin sections were deparaffinized with xylene, dehydrated with gradient ethanol, and washed with phosphate-buffered saline (PBS). For antigen retrieval, sections were immersed in ethylenediaminetetraacetic acid (EDTA) buffer (1:50, pH 9), kept in a preheated water bath at 60 °C and incubated overnight. A 30 min incubation was performed in 3% hydrogen peroxide to reduce nonspecific staining, followed by 30 min blocking with 5% goat serum at room temperature.

Sections were probed for 12 h at 4 °C with primary antibodies: anti-NF-κB P65 (ab32536), anti-iNOS (ab283655), anti-3-NT (ab61392), anti-CD63 (ab217345), anti-CD81 (ab109201), and anti-p-MLKL (ab196436) (all from Abcam). Sections were incubated with biotin secondary antibody for 1 h and then incubated with horseradish peroxidase for 30 min at room temperature. The target protein was stained with diaminobenzidine (DAB), and the nucleus with hematoxylin. Sections were observed and imaged by a fluorescence microscope (Olympus, BX63, Japan).

### Western blotting

Total protein was extracted from cerebral cortical tissue using a mammalian protein extraction reagent (Pierce, Rockford, IL, USA). Protein concentration was determined with a bicinchoninic acid (BCA) protein concentration assay kit, and 50 µg of the protein extract was loaded onto a sodium dodecyl sulfate–polyacrylamide gel and separated by sodium dodecyl sulfate–polyacrylamide gel electrophoresis (SDS-PAGE) in reducing conditions. The separated proteins were transferred to a polyvinylidene fluoride (PVDF) membrane. Membranes were blocked with nonfat dry milk to prevent nonspecific binding at room temperature overnight. The membrane was incubated with the following primary antibodies: anti-NF-κB P65 (ab32536), anti-iNOS (ab283655), anti-3-NT (ab61392), anti-CD63 (ab217345), anti-CD81 (ab109201), anti-TNF-α (ab1793), and anti-p-MLKL (ab196436) (all from Abcam); anti-Caspase-8 (13423–1-AP), anti-RIPK1 (17519–1-AP), and anti-RIPK3 (17563–1-AP) (Proteintech Group, Inc.). The membrane was probed with horseradish peroxidase-labeled secondary antibodies for chemiluminescent detection. The optical density values of the target bands were analyzed using a chemiluminescence imaging system.

### Enzyme-linked immunosorbent assay (ELISA)

Fresh cerebral cortical tissue was collected and stored at −80 °C until use. Protein levels of 4-HNE (E-EL-0128c) and 8-epi-PGF2α (E-EL-0041c) were quantified using ELISA kits as per the manufacturer’s recommendations.

### Reactive oxygen species (ROS) assay

The mouse cerebral cortex was prepared into single cell suspension by the mechanical method, and greater than 10^6^ cells were obtained. The Elabscience reactive oxygen species (ROS) fluorescence assay kit (green) was used. The collected cells were incubated with 10 µM diacetyldichlorofluorescein (DCFH-DA) probe at 37 ℃ for 30 min, washed three times with Dulbecco’s modified Eagle medium (DMEM) and resuspended in DMEM. The negative control was directly put on the machine without a probe, and the positive control was stimulated with 50 µM reagent II (positive control) for 2 h. After washing three times with DMEM, the cells were incubated with 10 µM DCFH-DA at 37 ℃ for 30 min before being tested by the machine. A Beckman Coulter CytoFlex SRT flow cytometer with a B525 emission filter was used and 10,0000 events were collected. The viable neurons were selected, and then the single cells were selected according to forward scatter/side scatter (FSC/SSC). The negative control cells without probes were detected by the computer (the gate was set according to the negative peak) and were detected after incubating the probes. DCFH-DA was used to evaluate the intracellular concentration of H_2_O_2_ as an index of ROS, and the content of ROS produced in each group was evaluated by counting the mean fluorescence intensity (MFI) of each group [39, 40].

### Statistical analysis

Data were analyzed with GraphPad Prism 8.1 software and expressed as the mean ± SEM. An unpaired *t*-test was used to compare the data corresponding to the single factor following the normal distribution between the two groups. Data corresponding to multiple factors were analyzed by two-way analysis of variance (ANOVA). Statistical significance was indicated when *P* < 0.05.

## Results

### Hypothermia causes necroptosis of cortical neurons

**Fig. 1:**
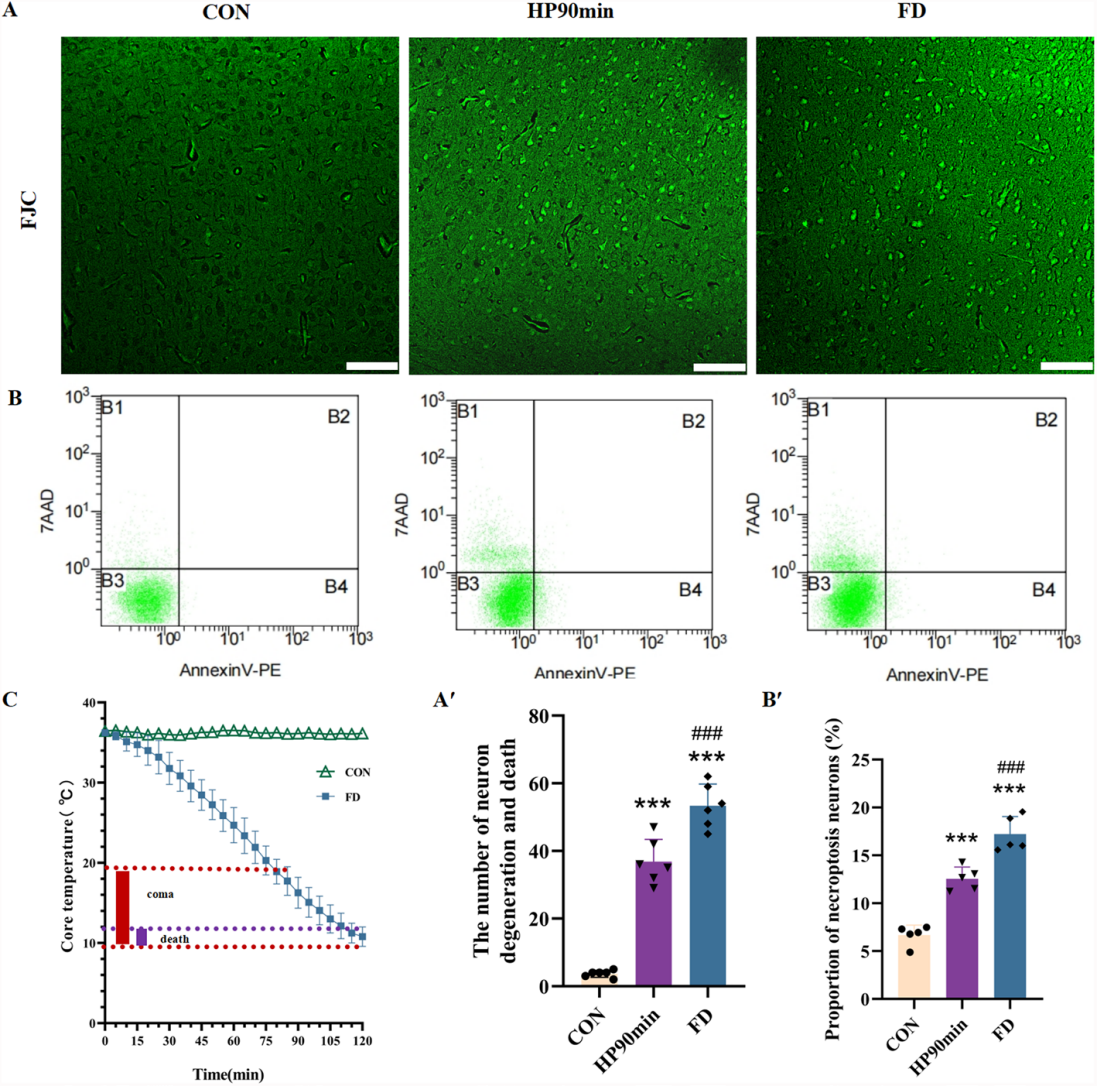
Hypothermia caused the degeneration and death of neurons in the cerebral cortex of mice. (**A**) Representative fluoro-jade C (FJC) images of cerebral cortical tissue. The degenerating and dead neurons are labeled with green fluorescence. Scale bars = 100 μm. (**A′**) Quantitative analysis of the numbers of degenerating and dead neurons in the cerebral cortex. (**B**) Representative flow cytometry images of cerebral cortical tissue. (**B′**) The percentage of necrotic apoptotic neurons in the cerebral cortex. (**C**) As the time in the 2–6 °C increased, the core body temperature of the mice gradually decreased. Data shown as means ± SEM; ***P < 0.001 versus the control group; ### *P* < 0.001 versus the HP90min group (*n* = 5)

The core body temperature of mice exposed to 2–6 ℃ ambient temperature, 65% humidity, and 0.2 m/s wind speed gradually decreased with prolonged exposure to the low temperature. The behavioral manifestations of mice progressed through several phases: motor active, resting tremor, coma, and death. The core body temperature of the coma mice was between 9.97 ℃ and 18.81 ℃, and that of the freezing-to-death mice was from 9.97 ℃ to 12.61 ℃ (Fig. 1C). Cerebral cortical tissue of mice was stained with FJC and the results showed that, compared with the control group, neuronal degeneration and death occurred in the cerebral cortex, especially in L5 pyramidal cells, but rarely in other parts of the cerebral cortex (Fig. 1A). In addition, the number of degenerated and dead neurons gradually increased with decreasing core body temperature. Cells in the cerebral cortex of mice were sorted with flow cytometry, revealing that hypothermia caused necroptosis in mouse cortical neurons compared with the control group (CON), and the proportion of necroptosis gradually increased with reducing core body temperature (Fig. 1B). These observations indicate that hypothermia causes degeneration and necroptosis of neurons in the cerebral cortex of mice. Moreover, hypothermia-induced necroptosis of cerebral cortical neurons is closely related to core body temperature and brain dysfunction.

### Characteristics of the necroptosis of cerebral cortical neurons caused by hypothermia

**Fig. 2:**
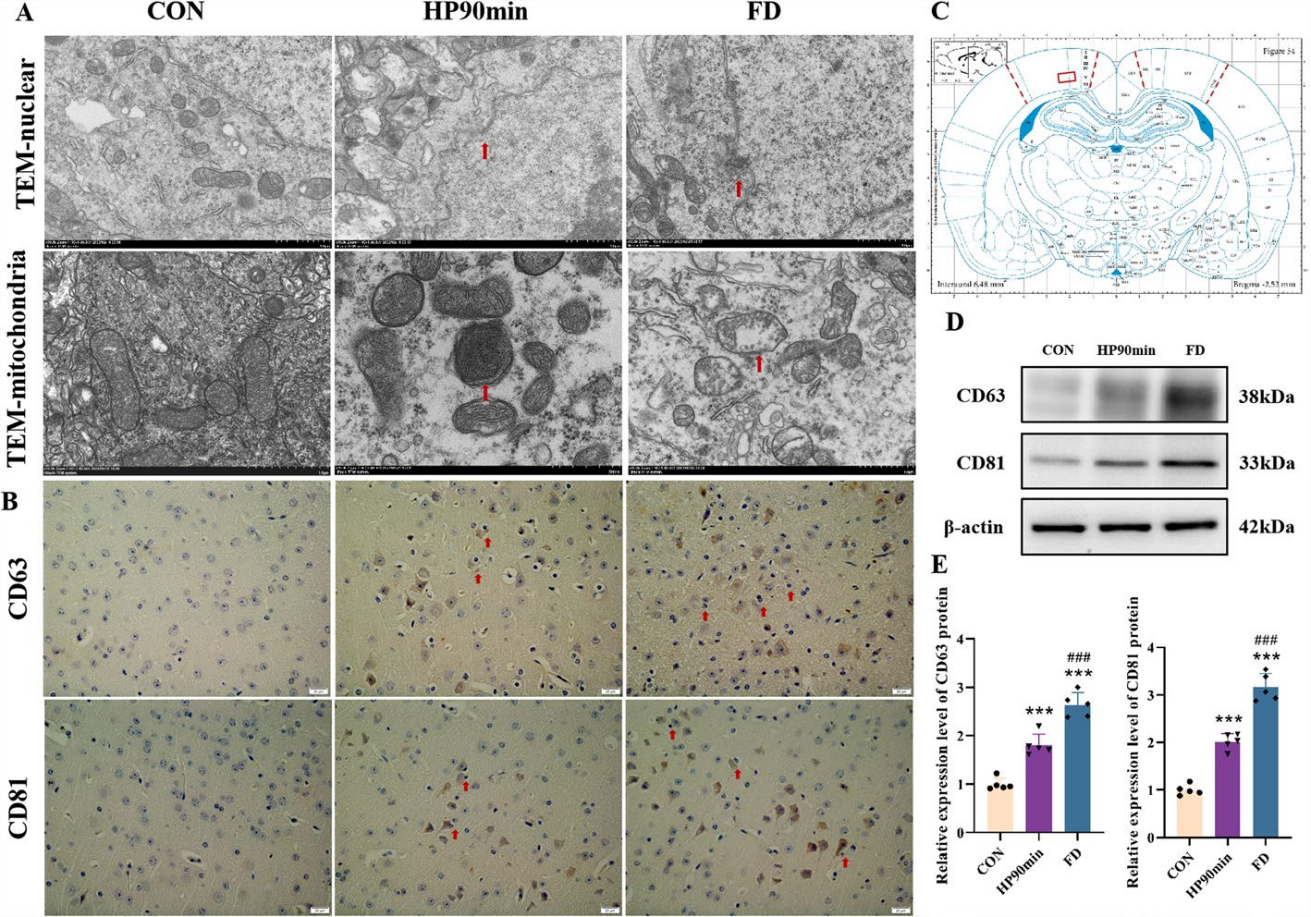
Characteristic changes in cerebral cortex neuronal cell death caused by hypothermia. (**A**) Representative transmission electron microscope (TEM) images of neuronal nuclei of the cerebral cortex. Scale bars = 1 μm. Hypothermia caused the rupture of the nuclear membrane of cerebral cortical neurons and the overflow of nuclear contents (red arrows, TEM-nuclear panels). Hypothermia also caused mitochondrial autophagy, swelling, and the disappearance of cristae (red arrows, TEM-mitochondria panels). (**B**) Representative immunohistochemistry (IHC) images of the cerebral cortex. CD63 and CD81 were expressed in the cytoplasm and nuclei of cortical neurons (shown as brown cells). The positive cells are cortical neurons, specifically in the pyramidal neurons of layer 5. The proliferation of microglia with neurophagocytosis can be seen around the positive cells (red arrows). Scale bars = 20 μm. (**C**) The immunohistochemical images of CD63 and CD81 were taken from the cerebral cortex as shown in (**C**) (diagram modified from Paxinos and Watson, 2007). (**D**) Expression of CD63 and CD81 in the cortex assessed by western blot. (**E**) Quantification of the protein levels in (**D**). Data shown as means ± SEM; ***P < 0.001 versus the control group; ###*P* < 0.001 versus the HP90min group (*n* = 5)

Ultrastructural changes in brain tissue often reflect the characteristics of hypothermia injury more clearly. Thus, we examined cerebral cortical tissue ultrastructure with transmission electron microscopy (TEM). It showed that hypothermia caused neuronal nuclear membrane rupture and heterochromatin leakage into the cytoplasm. It also uncovered partial mitochondrial swelling, membrane fusion, mitochondrial disruption and mitophagy, and enlarged rough endoplasmic reticulum with the shedding of bound ribosomes (Fig. 2A). These ultrastructural changes are consistent with the morphological characteristics of necroptosis [41–43], with prominent mitochondrial damage and nuclear membrane rupture.

Fibroblast death and the production of numerous exosomes under combined low temperature and ultraviolet light irradiation [44, 45] are unique manifestations previously unreported in forensic pathology practice. The immunohistochemical examination showed that CD63 and CD81 exosome marker proteins were localized in the neurons of the cortical tissue, especially in L5 pyramidal neurons. Western blotting showed that the levels of CD63 and CD81 proteins in the cerebral cortex were significantly upregulated under hypothermia compared with the CON group, and gradually increased with decreasing core body temperature (Fig. 2B-C). These results indicate that hypothermia induces a surge of exosomes from cerebral cortical neurons, providing a potential marker for identifying death from freezing temperatures in forensic pathology. They also show that hypothermia causes necroptosis of cerebral cortical neurons, characterized by apparent mitochondrial damage, nuclear membrane rupture, and nuclear content overflow.

### Hypothermia causes transcriptomic and metabolomic changes in mouse cerebral cortical tissues

#### Transcriptome profile

**Fig. 3:**
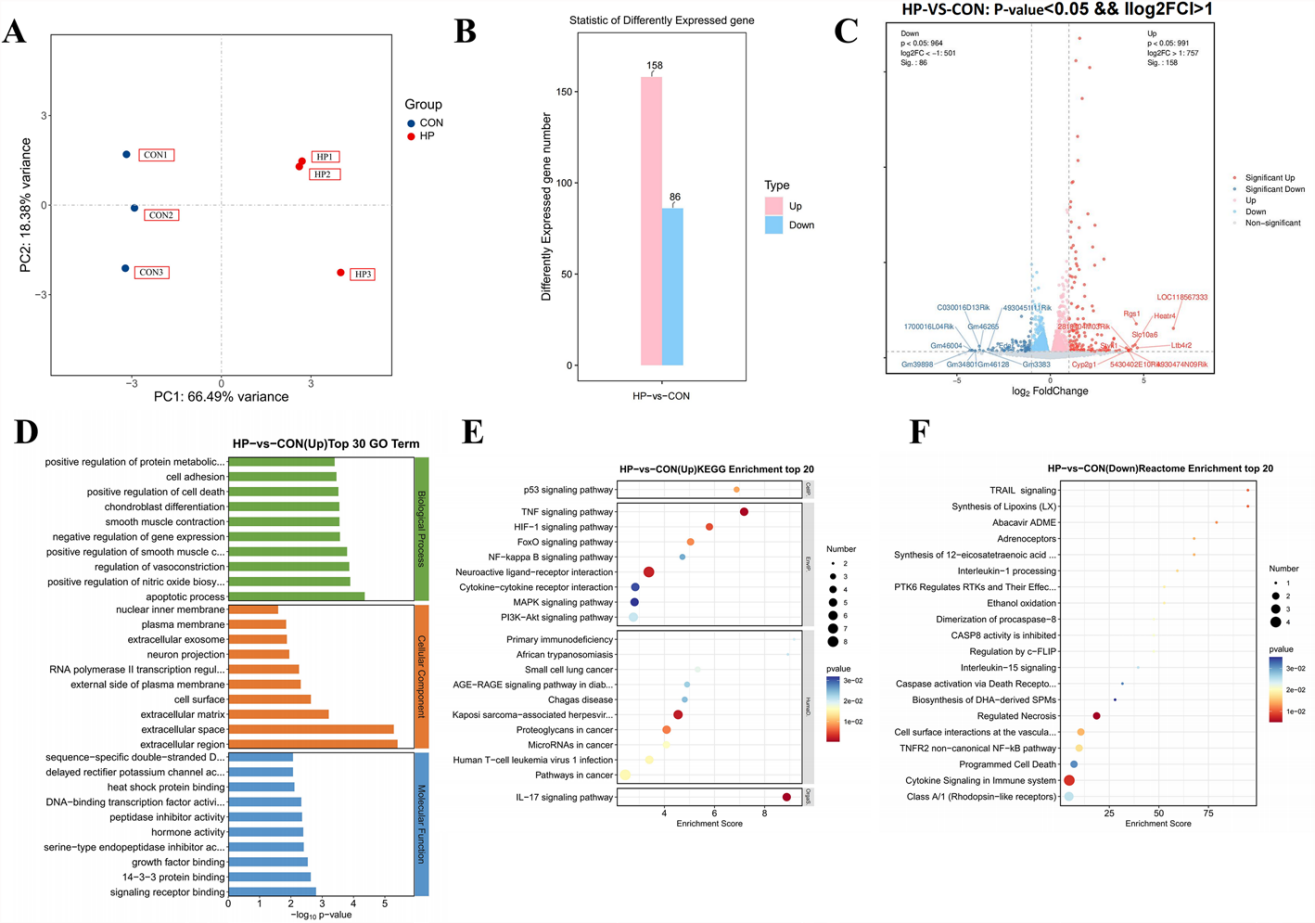
Volcano and bubble plots of transcriptomic changes in the cerebral cortex caused by hypothermia. (**A**) The quality of the principal component analysis (PCA) score plot. All QC samples clustered together, suggesting that the collection and analysis of the data were reliable, stable, and correct. (**B**) Bar graphs of the statistics of the differentially expressed genes in the hypothermia (HP) versus control (CON) groups. The pink column represents the number of upregulated genes with significant differences. The blue column represents the number of downregulated genes with significant differences. (**C**) Volcano plot of differentially expressed genes in the HP versus CON groups. In the volcano plot, the red dots represent upregulated genes, the blue dots represent downregulated genes, and the grey dots represent no significant difference (namely, genes that are detected but did not meet the filtering parameters for screening). The R language “limma” package was applied to identify differentially expressed genes. (**D**) Bubble chart of Gene Ontology (GO) enrichment top 30 in HP versus CON groups. (**E**) Bubble chart of the Kyoto Encyclopedia of Genes and Genomes (KEGG) enrichment top 20 in HP versus CON groups. The size of the dots in the graph represents the number of significantly different genes enriched in the corresponding pathways. (**F**) Bubble chart of reactome enrichment top 20 in HP versus CON groups. The size of the dots in the graph represents the number of significantly different genes enriched in the corresponding pathways

Transcriptomic studies reveal the mechanisms of hypothermia-induced neuronal cell death in the mouse cerebral cortex and provide targets for the diagnosis and treatment of hypothermia coma. We sequenced the transcriptome of mouse cerebral cortical tissue between the HP90min (hypothermia coma) and control groups to investigate target genes regulating hypothermia injuries. A principal component analysis of the transcriptome samples showed a good separation between the HP90min and control groups, indicating that hypothermia causes substantial transcriptomic changes in the cerebral cortical tissue (Fig. 3A). Differentially expressed protein-coding genes were detected by standardizing the number of genes in each sample (with base mean value estimating the expression level) and calculating the fold change with DESeq2 software. In addition, a negative binomial distribution test was used to assess significant differences between two samples and recognize the genes using the calculated significance and fold change. A total of 244 differentially expressed genes were identified between the HP90min and control groups, with 158 upregulated and 86 downregulated genes (Fig. 3B). These genes were subjected to a Gene Ontology (GO) enrichment analysis to infer their biological functions (Fig. 3D). The top 30 genes significantly upregulated by hypothermia were enriched in several GO biological processes: apoptotic processes, positive regulation of nitric oxide biosynthesis, and positive regulation of cell death. These data imply that the genes most induced by hypothermia in the mouse cerebral cortex are related to cell death and nitric oxide production. Furthermore, a KEGG enrichment analysis of the differential genes showed that they were most enriched in P53, TNF, HIF-1, FoxO, and NF-kappa B signaling pathways (Fig. 3E). Hence, these genes are associated with signaling pathways possibly involved in hypothermia-induced neuronal cell death in the mouse cerebral cortex.

#### Metabolome profile

**Fig. 4:**
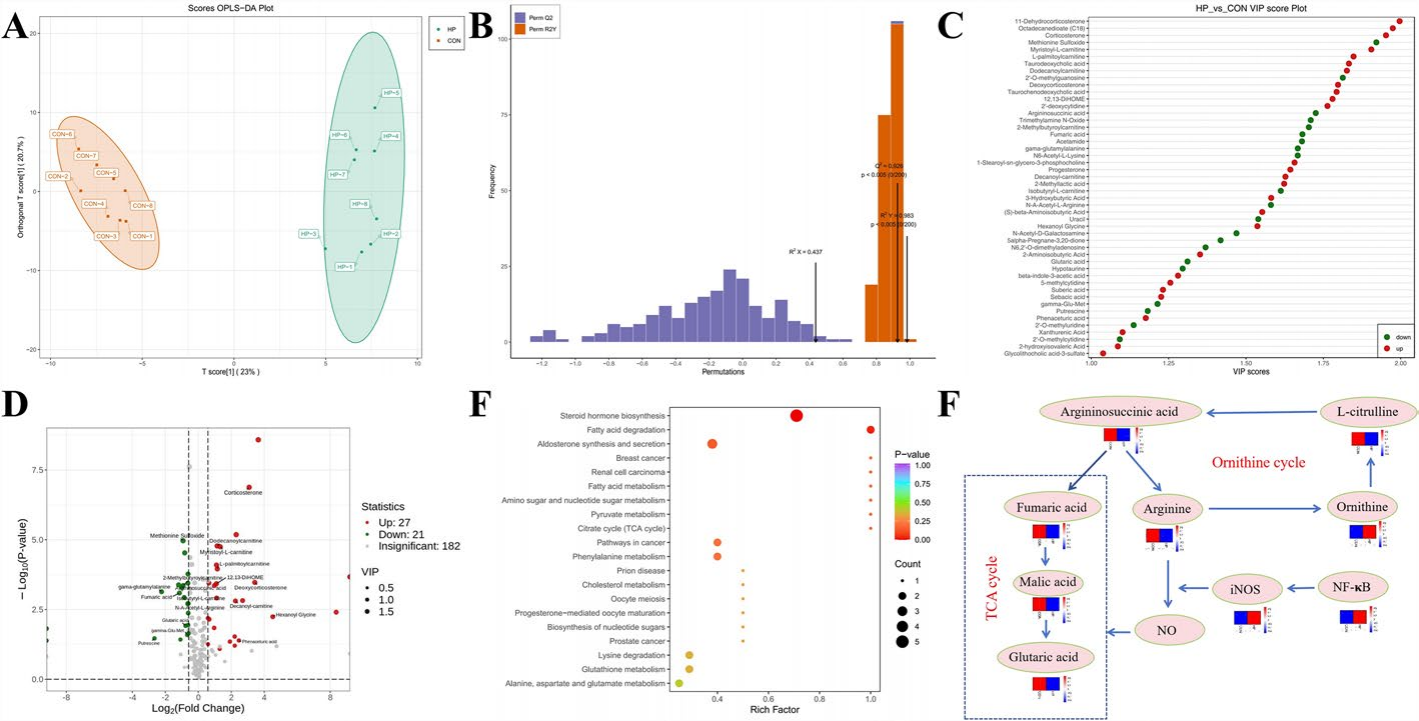
Hypothermia causes metabolomic changes in the cerebral cortex. Integration analysis with transcriptomics. (**A**) Score plot of the orthogonal partial least squares discriminant analysis (OPLS-DA) model in HP versus CON groups. Orange represents the control group, and green represents the HP group. (**B**) OPLS-DA permutation test. Orange represents the random grouping model R^2^Y, purple represents the random grouping model Q^2^, and black arrows represent the values for the R^2^X, R^2^Y, and Q^2^ values of the original model. (**C**) VIP score plot of the OPLS-DA. The VIP in the OPLS-DA model was a predominant parameter for the detection of potential differential metabolites. VIP score plot of metabolites verified in the OPLS-DA model between the HP and CON groups. Red and blue represent the high and low relative contents of the corresponding metabolites. (**D**) Volcano plot of HP versus CON groups. In the volcano plot, the red dots represent upregulated metabolite, the blue dots represent downregulated metabolites, and the grey dots represent no significant difference (namely, metabolites that are detected but did not meet the filtering parameters for screening). The R language “limma” package was applied to identify differentially expressed metabolites. (**E**) Bubble chart of KEGG enrichment in HP versus CON groups. The closer the *P*-value is to 0, the more significant the enrichment. The size of the dots in the graph represents the number of significantly different metabolites enriched in the corresponding pathways. (**F**) Schematic representation of the integrated analysis of metabolomic and transcriptomic significant differences. Circles represent metabolites or genes, with gene names written in italics and metabolite names in normal font. The values in the heatmap represent the *z*-score of the fragments per kilobase of exon per million mapped fragments (FPKM) values (transcript or metabolic levels) in different samples. Red color indicates a high expression level, while blue is low

Metabolic changes of substances are regulated by the transcriptome (the sum of all RNAs transcribed from the genome). The cerebral cortical tissue metabolic profile was determined using extensively targeted metabolomics to analyze the relationship between hypothermia and the discovered metabolites. In orthogonal partial least squares discriminant analysis, a good separation between the HP90min and control groups was observed (Fig. 4A), suggesting that hypothermia causes severe metabolic dysfunction in the cerebral cortex. In addition, 200 permutation tests (R^2^Y = 0.983, Q^2^ = 0.926, *P* < 0.005) showed that the orthogonal partial least squares discriminant analysis model had reliable predictive power (Fig. 4B). Furthermore, the s-plot derived from the model revealed differences in the expression of metabolites with variable importance in the projection (VIP) values > 1 or < 1 between the HP90min and control groups. Volcano plots were used to identify differential metabolites from all detected metabolites after hypothermia exposure on the basis of VIP values and fold changes (VIP > 1.0, fold change > 2/ < 0.5) (Fig. 4D). A total of 48 differential metabolites were detected between the hypothermia coma and control groups, including 21 metabolites with decreased levels and 27 with increased levels (Fig. 4C). Among the differential metabolites, hormones and hormone-related compounds such as corticosterone and deoxycorticosterone, significantly increased in the HP90min group versus the control group, indicating a considerable elevation of catecholamine hormones under low-temperature stress. In contrast, amino acid derivatives such as *N*-acetyl-L-arginine, argininosuccinic acid, glutaric acid, and fumaric acid significantly decreased. L-palmitoylcarnitine, myristoyl-L-carnitine, dodecanoylcarnitine, and other acylcarnitines were also significantly elevated. Finally, a pathway enrichment analysis using the KEGG database was performed to infer the pathways associated with the differential metabolites (Fig. 4E). Among all enriched pathways, those related to amino acid and fatty acid metabolism were the most abundant and caused significant changes in the TCA cycle.

#### Integrated analysis of metabolomic and transcriptomic data

Transcriptomic and metabolomic results were integrated to suggest the mechanism of hypothermia-induced necroptosis of cortical neurons in the mouse cerebrum. The integrated analysis (Fig. 4F) found that hypothermia may activate nuclear factor of kappa light polypeptide gene enhancer in B cells 1 (*NF-κB*), which, in turn, activates inducible nitric oxide synthase (*iNOS*) to consume arginine and produce nitric oxide (NO), compromising the ornithine cycle, owing to excessive arginine depletion. These data indicate that excessive NO damages mitochondria through oxidative stress, disordering the TCA cycle and eventually provoking necroptosis of cerebral cortical neurons in hypothermia-exposed mice.

### Hypothermia-induced NF-κB activation triggers oxidative stress and TNF-α leading to neuronal necroptosis

**Fig. 5:**
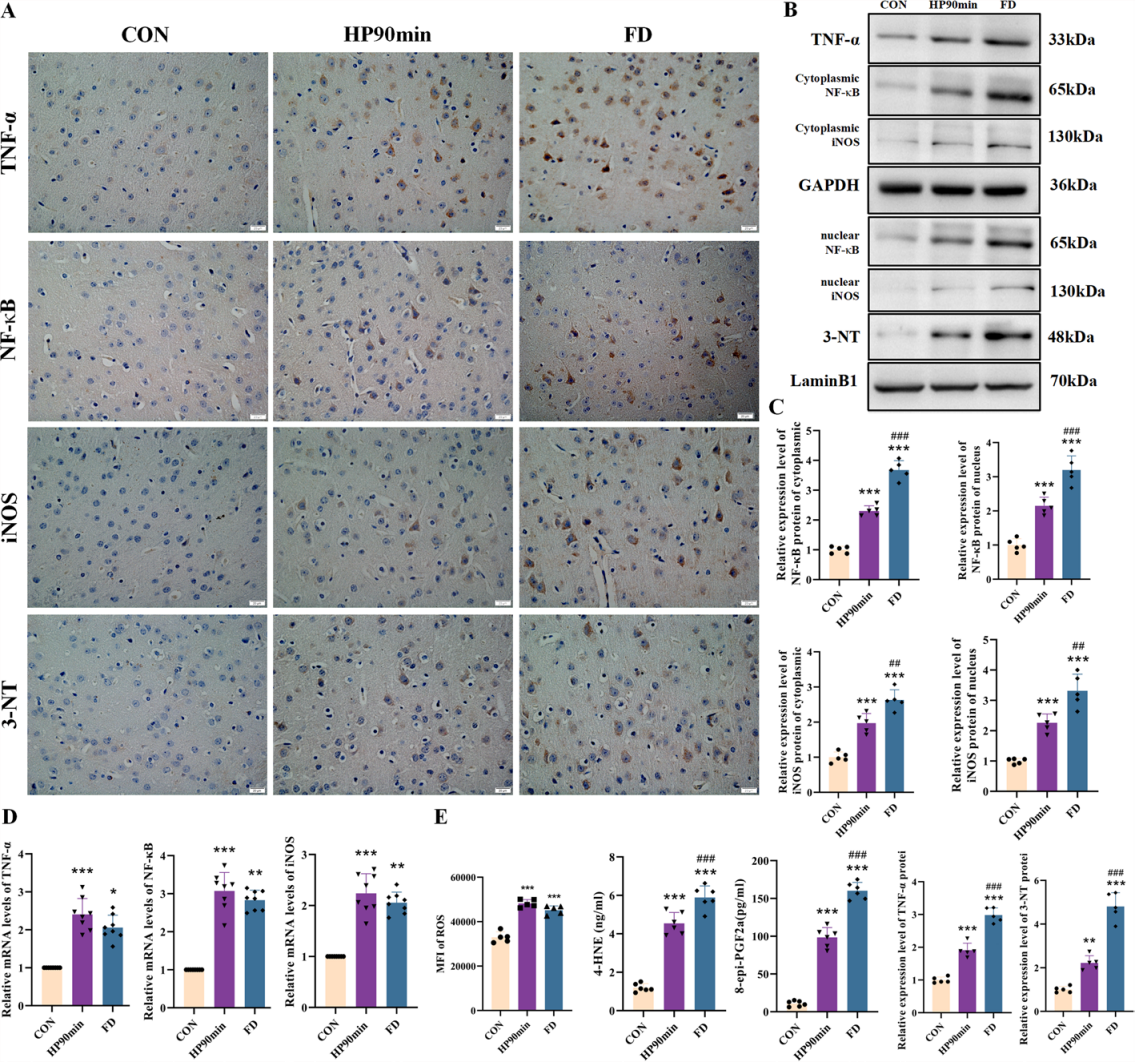
The expression of tumor necrosis factor (TNF)-α, nuclear factor (NF)-κB, inducible nitric oxide synthase (iNOS), and 3-nitrotyrosine (3-NT) in the cerebral cortex caused by hypothermia. (**A**) Representative immunohistochemistry (IHC) images of neurons of the cerebral cortex. The IHC images within the cerebral cortex are schematically illustrated in Fig. 2C. TNF-α, NF-κB, iNOS, and 3-NT were expressed in the cytoplasm and/or nuclei of cortical neurons (positive cells are brown). The positive cells were cortical neurons, specifically in the pyramidal neurons of layer 5. Scale bars = 20 μm. (**B**) Expression of TNF-α, NF-κB, iNOS, and 3-NT in the cortex assessed by western blot. (**C**) Quantification of the protein levels in (**B**). (**D**) Relative mRNA levels of *TNF-α*, *NF-κB*, and *iNOS* in the cerebral cortex of mice. (**E**) Levels of ROS, 4-hydroxynonenal‌ (4-HNE), and 8-epi-PGF2α in the cerebral cortex of mice. Data shown as means ± SEM; *P < 0.05, **P < 0.01, ***P < 0.001 versus the control group; ## *P* < 0.05, ### *P* < 0.001 versus the HP90min group (*n* = 5)

The NF-κB-induced oxidative stress and TNF-α-induced necroptosis pathways were explored to verify the integrated omics results. The RT-PCR examination of the cortical tissue showed that hypothermia caused a significant increase in tumor necrosis factor alpha (*TNF-α*), *NF-κB*, and *iNOS* gene expression (Fig. 5C). In addition, TNF-α, NF-κB, iNOS, and 3-NT protein expression was localized in the cytoplasm and nuclei of cerebral cortical neurons, especially in L5 pyramidal neurons (Fig. 5A). A western blotting analysis showed that hypothermia significantly elevated TNF-α, NF-κB, iNOS, and 3-NT protein expression in cortical neurons compared with the CON group, in agreement with the induced expression of these genes (Fig. 5B). Low temperature stress stimuli activate NF-κB and iNOS proteins to produce an excess amount of NO [45], which reacts with superoxide anions (O_2_^−^) to generate peroxynitrite anions (ONOO^−^). This anion reacts with numerous proteins since it induces nitration of protein-bound or free tyrosine residues to form 3-nitrotyrosine (3-NT), triggering vigorous oxidative stress. Indeed, the ELISA results confirmed that the cortical tissue exposed to hypothermia exhibited induced expression of 4-hydroxynonenal‌ (4-HNE), and 8-epi-PGF2α compared with the CON group, known oxidative stress biomarkers. At the same time, the study found that low temperatures caused the cerebral cortex tissue to produce a large amount of ROS (Fig. 5E).

**Fig. 6:**
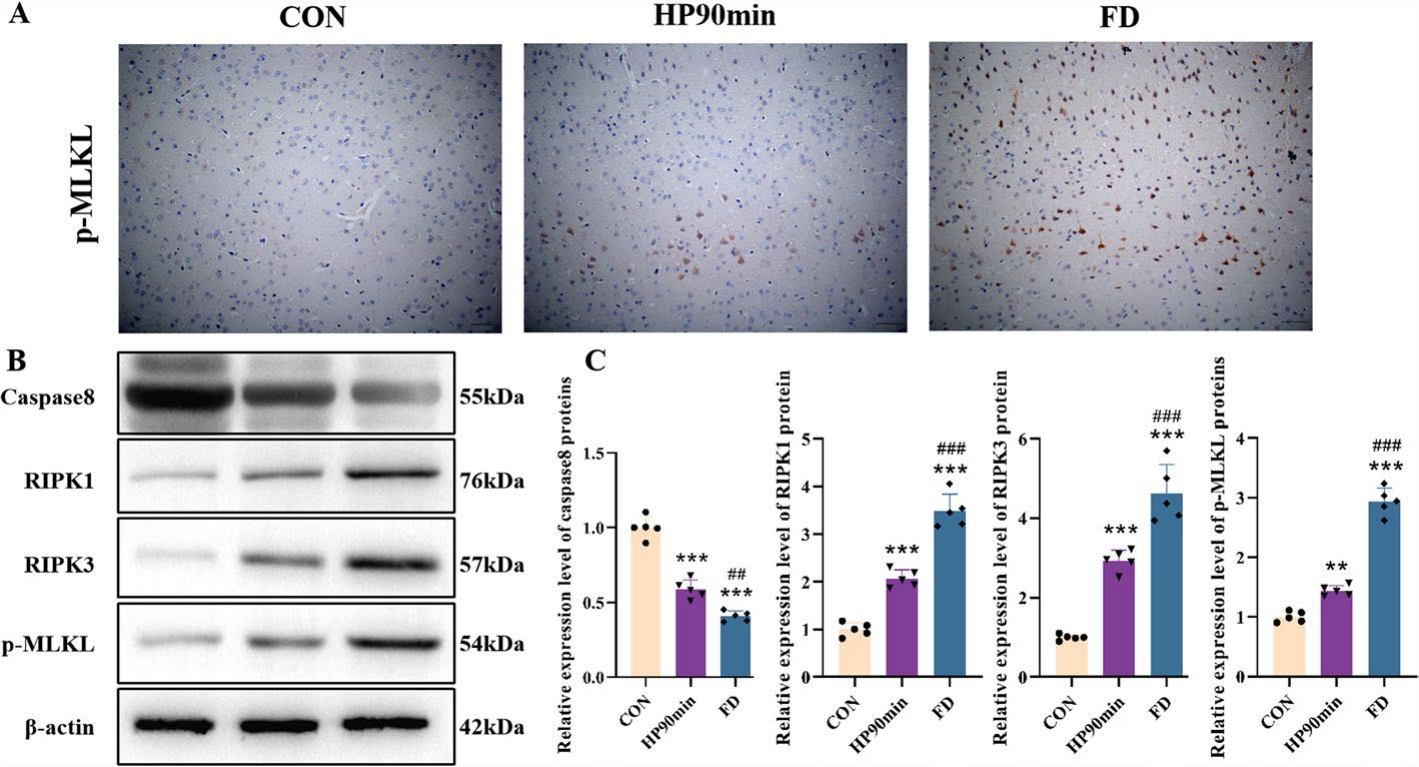
The expression of caspase 8, RIPK1, RIPK3, and p-MLKL in the cerebral cortex caused by hypothermia. (**A**) Representative IHC images of neurons of the cerebral cortex. p-MLKL was expressed in the cytoplasm and nuclei of cortical neurons (positive cells are brown). The positive cells were cortical, specifically the pyramidal neurons of layer 5. Scale bars = 20 μm. (**B**) Expression of caspase 8, receptor-interacting serine/threonine protein kinase 1 (RIPK1), RIPK3, and phosphorylated mixed lineage kinase domain-like protein (p-MLKL) in the cortex, as assessed by western blot. (**C**) Quantification of the protein levels in (**B**). Data shown as means ± SEM; **P < 0.01, ***P < 0.001 versus the control group; ##*P* < 0.05, ###*P* < 0.001 versus the HP90min group (*n* = 5)

In addition to promoting oxidative stress damage in cells by activating iNOS, the NF-κB protein plays a pivotal role in necroptosis. Upon activation of death receptors, cellular inhibitor of apoptosis proteins (cIAPs) and Fas-associated death domain (FADD) activate NF-κB [46]. Activated NF-κB also promotes the release of the downstream proinflammatory cytokine TNF-α, a potent inducer of necroptosis. The western blot analysis results showed that hypothermia significantly increased receptor-interacting serine/threonine protein kinase 1 (RIPK1), RIPK3, and phosphorylated mixed lineage kinase domain-like protein (p-MLKL) levels, and decreased caspase 8 in the cerebral cortex (Fig. 6). These findings suggest that hypothermia induces oxidative stress by activating the NF-κB transcription factor in the mouse cerebral cortex, suggesting the mechanism of neuronal necroptosis.

### Inhibition of NF-κB alleviates neuronal necrosis and oxidative stress caused by hypothermia

**Fig. 7:**
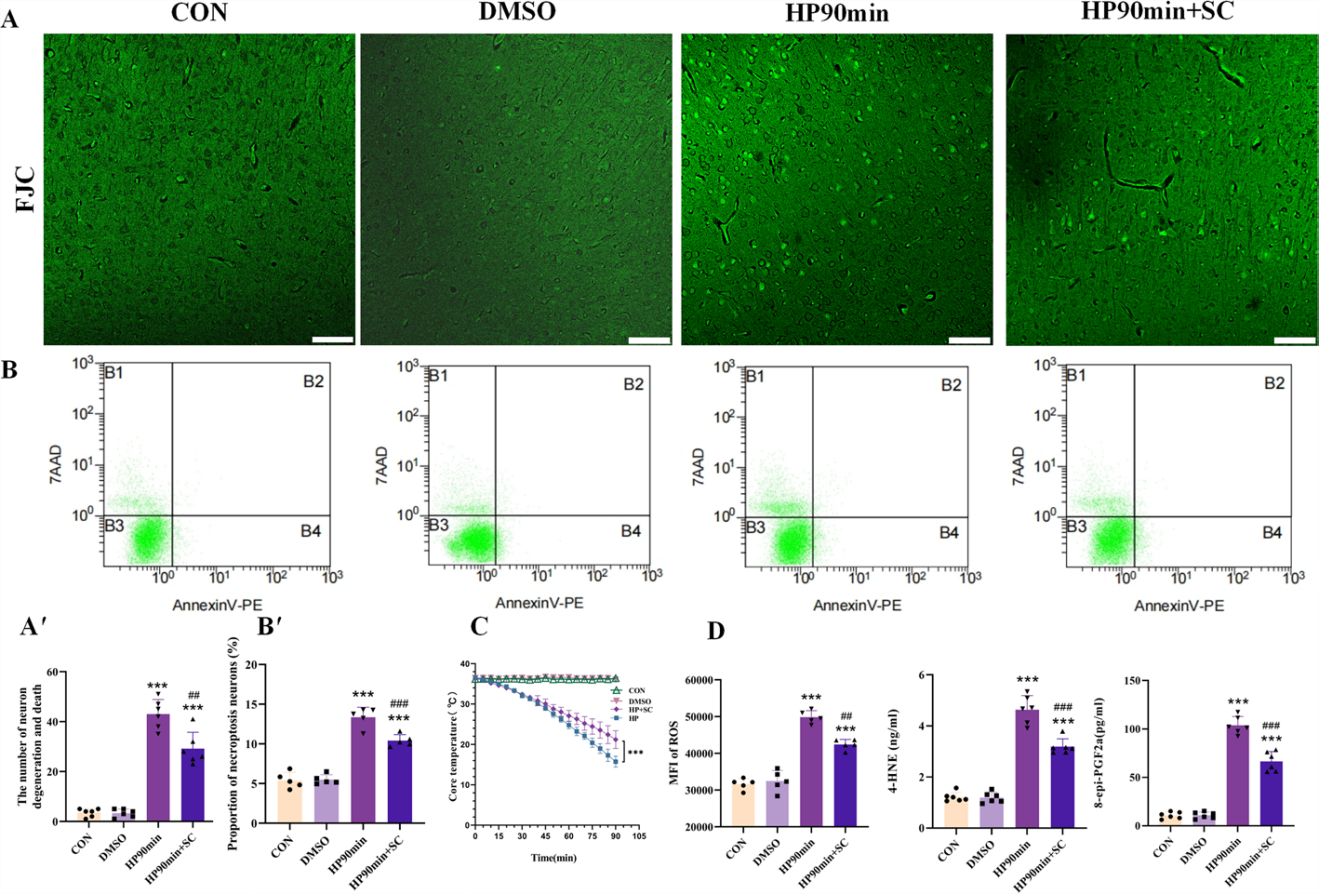
Inhibition of the NF-κB–iNOS pathway alleviated the degeneration and death of neurons in the cerebral cortex after hypothermia. (**A**) Representative FJC images of the cerebral cortex of mice. The positive cells were labeled with green fluorescence. Scale bars = 75 μm. (**B**) Representative flow cytometry images of cerebral cortical cells. (**A′**) Quantitative analysis of the positive cell number in the cerebral cortex. (**B′**) The percentage of necrotic apoptotic neurons in the cerebral cortex. (**C**) As the time at 2–6 °C increased, the core body temperature of the mice gradually decreased in HP and HP + SC groups. (**D**) Levels of ROS, 4-HNE, and 8-epi-PGF2α in the cerebral cortex of mice. Data shown as means ± SEM; **P < 0.01, ***P < 0.001 versus the control group; ##*P* < 0.05, ###*P* < 0.001 versus the HP90min group (*n* = 5)

The role of NF-κB in hypothermia-induced necroptosis of cerebral cortical neurons was evaluated using a potent NF-κB inhibitor, SC75741 (1 mg/kg). It decreased the expression of downstream iNOS by impairing DNA binding of NF-κB subunit p65, relieving oxidative stress damage [29, 47, 48]. The effect of NF-κB inhibition on hypothermia-induced neuronal injury was investigated. The results showed that the SC75741 inhibitor effectively reduced the expression of 4-HNE and 8-epi-PGF2α stress biomarkers, as well as the proportion of cell degeneration and necroptosis in cerebral cortical neurons caused by hypothermia (Fig. 7A, B, D). The results also showed that pharmacological inhibition of NF-κB improves mental integrity and core body temperature of mice exposed to low temperatures (Fig. 7C). These results indicate that oxidative stress damage triggered by NF-κB is a key mechanism by which hypothermia-induces necroptosis in cerebral cortical neurons.

## Discussion

On the basis of the degree of influence of body temperature on the human body and different physiological responses, core body temperature is typically divided into mild (32–35 ℃), moderate (28–32 ℃), and severe hypothermia (< 28 ℃)[49]. Our study discovered that as the mice spent more time in the low temperature, their core body temperature gradually decreased until they died. The core body temperature of hypothermia coma mice was 9.97–18.81 °C, and that of freezing-to-death mice was 9.97–12.61 °C, indicating that the lower the core body temperature, the more serious the damage to the body.

Severe hypothermia triggers neurological dysfunction, primarily by affecting ion channel gating mechanisms or the inability of lipid membranes to maintain ionic homeostasis and depolarization of cell membranes, leading to loss of neuronal excitability [50]. For example, severe hypothermia (< 15 °C) causes brain swelling, Na^+^ accumulation in the brain parenchyma, and K^+^ depletion. These events may be caused by a mismatch between active and passive membrane transport processes, leading to membrane depolarization and irreversible cell damage [51, 52]. In this study, flow cytometry, FJC staining, and TEM analyses confirmed that severe hypothermia causes necroptosis of cerebral cortical neurons, especially L5 pyramidal cells. Excitatory pyramidal cells are crucial for maintaining the functions of the cerebral cortex. Pyramidal cell death decreases or abolishes the excitability of the cerebral cortex, which may also be a decisive factor causing hypothermia-induced coma or death. Hypothermia may lead to cognitive impairment, and necroptosis is a central mechanism in the pathogenesis of this disease [53, 54]. Combined exposure to low temperatures and ultraviolet light provokes the death of fibroblasts and other cells, accompanied by elevated exosome production [44, 45]. Our data suggest that hypothermia triggers neuronal necroptosis via enhanced exosome release from cortical neurons, which may be one of the major characteristics of hypothermia injury.

Specific core body temperature causes unique metabolic changes in brain tissue. Extensively targeted metabolomic analysis of the cerebral cortical tissues showed that severe hypothermia elevated the levels of stress hormones, such as corticosterone and deoxycorticosterone, and long-chain acylcarnitines. Conversely, hypothermia decreased arginine, argininosuccinic acid, fumaric acid, and glutamate levels. Moreover, the enrichment analysis showed significantly compromised fatty acid and TCA metabolism. Lower core body temperature increases the levels of catecholamines and glucocorticoids by stimulating the hypothalamic–pituitary–adrenal axis [55–58]. Activation of the sympatho-adrenal system, which enhances lipolysis and increases free fatty acid levels in the blood, is one of the major mechanisms for maintaining thermal homeostasis [59, 60]. Free fatty acids and ketone bodies represent the main energy substrates for neural tissue metabolism; therefore, they counteract the effects of body cooling. However, their protective function is disrupted under severe hypothermia owing to mitochondrial damage that impairs long-chain/short-chain fatty acid metabolism, accumulating long-chain fatty acids/acylcarnitine. Palmitic, stearic, and oleic acids are considered potential markers of fatal hypothermia [61].

Various core body temperatures have diverse transcriptional effects on the brain tissue. Our transcriptomic analysis of cerebral cortical tissues showed that severe hypothermia activated NF-κB signaling, NO metabolism, and the necroptosis pathway, and inhibited caspase 8. Argininosuccinate lyase breaks down argininosuccinate to fumarate and L-arginine, which in turn generates NO and L-citrulline in response to iNOS [62–64]. Limited L-arginine levels activate iNOS to generate large amounts of NO, disturbing the ornithine cycle and hampering glutamate production. Glutamate has a general and powerful excitatory effect on cerebral cortical neurons, and activated glutamate receptors participate in rapid excitatory synaptic transmission. Glutamate also participates in apoptosis, Ca^2+^ influx, calmodulin binding, nitric oxide synthase binding, NO synthesis, enzyme activity regulation, and normal DNA synthesis and repair [65]. Therefore, hypothermia-induced ornithine cycle disruption may also be among the mechanisms causing cortical neuron death and central nervous system paralysis.

Multiomic combination analysis can more comprehensively and accurately reveal the mutual influence and regulatory relationship between diverse types of molecules and identify the key molecules and pathways of necroptosis in cerebral cortical neurons affected by severe hypothermia [66]. Our combined metabolomic and transcriptomic data analysis suggests severe hypothermia activates the NF-κB transcription factor. Subsequently, it activates iNOS to consume arginine and accumulate NO, impeding the ornithine cycle owing to excessive arginine consumption. Excessive NO levels cause mitochondrial damage through oxidative stress, destroying the TCA cycle and eventually provoking necroptosis of cerebral cortical neurons. Under mild hypothermia, NO production by the nervous system indirectly provides substrates for mitochondrial respiration by dilating blood vessels and regulating blood flow in tissues. It also protects neurons by regulating mitochondrial pH, membrane potential, the respiratory chain, and energy production [17]. However, excess NO damages mitochondria through the oxidative stress pathway, compromising cellular energy metabolism [67]. Accumulated NO reacts with O_2_^−^ generated during oxidative phosphorylation to form ONOO^−^, which destroys key mitochondrial components in the matrix, inner and outer membranes, and the intermembrane space. In mitochondria, ONOO^−^ inhibits I, II, and IV complexes in the respiratory chain and ATP synthase activity, and also affects the activity of other proteins, such as manganese superoxide dismutase (MnSOD) and cytochrome C, causing mitochondrial dysfunction. Combined hypothermia and ultraviolet light conditions activate iNOS, which releases numerous NO bubbles, causing nuclear rupturing and, eventually, cell death [44, 45]. ROS scavengers strongly inhibit TNF-α-induced necroptosis. During necroptosis, mitochondrial ROS levels increase, depolarization occurs, and the ATP concentration decreases [68]. Oxidative stress is increasingly recognized as a driving force for necroptosis. In summary, our multiomics data support oxidative stress as one of the important driving factors for hypothermia-initiated necroptosis in the mouse cerebral cortex.

The TNF-α protein is a potent necroptosis inducer [69] that binds to tumor necrosis factor receptor 1 on the cell membrane surface to initiate its conformational change. Consequently, the activated receptor forms a complex with TNFRSF1A-associated via death domain and RIPK1 [70]. When ATP is scarce, caspase 8 activity is inhibited, allowing RIPK1 and RIPK3 to interact through their RIP homologous interaction domains and form the RIPK1–RIPK3 necrosis complex or necrosome [41]. In the necrosome, RIPK1 phosphorylates and activates RIPK3, which phosphorylates mixed lineage kinase domain-like (MLKL) at positions T357 and S358. As a result, a conformational shift in MLKL exposes its 4-helix bundle domain, followed by MLKL oligomerization and translocation to the plasma membrane through its N-terminal domain. Finally, MLKL directly or indirectly destroys the plasma membrane, driving necroptosis [41]. Our study found that severe hypothermia caused a significant increase in the expression of necroptosis-related proteins in cerebral cortical neurons, accompanied by a significant microglial proliferation. Moreover, FJC staining of the cortex showed that hypothermia-induced necroptosis was mainly distributed in L5 pyramidal cells. This revelation is intriguing and may be closely related to calcium/calmodulin-dependent protein kinase II (CaMKII), a novel RIPK3 substrate that binds RIPK3 to promote the opening of mitochondrial permeability transition pore (mPTP), mediating necroptosis via the RIPK3–CaMKII–mPTP signaling pathway [71].

Thus, evidence points to an interaction between oxidative stress and necroptosis. Abundant ROS levels promote the stabilization of the RIPK1/RIPK3 necrosome complex, driving necroptosis [72]. Reducing ROS levels by ROS scavenger application blocks the interaction between RIPK1 and RIPK3 [73], repressing necroptosis. Similarly, H_2_O_2_ induces necroptosis of rat nucleus pulposus cells, and pretreating them with necroptosis inhibitor Nec-1 alleviates the H_2_O_2_-induced necroptosis. Apart from ROS, proteins in the necroptosis pathway influence the occurrence of oxidative stress [74]. The RIPK3 protein is one of the crucial proteins in the necroptosis pathway since it modulates TNF-α-induced ROS production [75]. ROS production is suppressed upon TNF stimulation, and Nec-1 pretreatment reduces the expression levels of RIPK1/RIPK3 [76]. Thus, a positive feedback amplification loop exists between RIPK1/RIPK3 and ROS to regulate TNF-α-induced necroptosis. In addition, MLKL may also transfer to the mitochondrial membrane, causing excessive opening of the mPTP and excessive ROS production, which in turn, further aggravates cell death [77]. In this study, we found that pharmacological inhibition of NF-κB with the SC75741 inhibitor significantly attenuated necroptosis in hypothermia-exposed cerebral cortical neurons. The connection between oxidative stress and necroptosis through the TNF-α–NF-κB pathway is also known from studies on cadmium exposure [69]. Therefore, oxidative stress and necroptosis have a mutual promoting effect and are both actively involved in the injury of cerebral cortical neurons under severe hypothermia.

## Conclusions

Our study reveals that severe hypothermia activates the NF-κB–iNOS signaling pathway, depletes arginine to produce NO, and disrupts the ornithine cycle owing to excess arginine depletion. Excess NO damages mitochondria through oxidative stress, impairing the TCA cycle, which eventually causes necroptosis of cerebral cortical neurons and central nervous system paralysis. Our study provides potential evidence for the clinical treatment of hypothermia-induced coma. In addition, necroptosis induced by massive exosome release from severe hypothermia-exposed cortical pyramidal neurons is a potential indicator of fatal hypothermia in forensic pathology. This research provides valuable insights for coping with climate anomalies.

## Supplementary information

Additional file 1.

## Data Availability

The raw data supporting the conclusions of this article will be made available by the authors (17800584@hebmu.edu.cn) without undue reservation.

## Abbreviations

TNF-α: Tumor necrosis factor alpha
TCA cycle: Tricarboxylic acid cycle
FJC: Fluoro-jade C
NF-κB: Nuclear factor kappa-B
TEM: Transmission electron microscopy
MWDB: Metware database
LC–ESI–MS: Liquid chromatography–electrospray ionization–tandem mass spectrometry
KEGG: Kyoto Encyclopedia of Genes and Genomes
GSEA: Gene set enrichment analysis
RT-qPCR: Reverse transcription-quantitative PCR
PBS: Phosphate-buffered saline
EDTA: Ethylenediaminetetraacetic acid
SDS-PAGE: Sodium dodecyl sulfate–polyacrylamide gel electrophoresis
PVDF membrane: Polyvinylidene fluoride membrane
ELISA: Enzyme-linked immunosorbent assay
iNOS: Inducible nitric oxide synthase
3-NT: 3-Nitrotyrosine
ONOO^−^: Generate peroxynitrite anion
O_2_^−^: Superoxide anion
4-HNE: 4-Hydroxynonenal
8-epi-PGF2α: 8-Epi-prostaglandin F2alpha
cIAPs: Cellular inhibitor of apoptosis proteins
FADD: Fas-associated death domain
RIPK1: Receptor-interacting serine/threonine protein kinase 1
RIPK3: Receptor-interacting serine/threonine protein kinase 3
p-MLKL: Phosphorylated mixed lineage kinase domain-like protein
ROS: Reactive oxygen species
ATP: Adenosine-triphosphate
CaMK II: Calcium/calmodulin-dependent protein kinase II
mPTP: Mitochondrial permeability transition pore
